# The Role of Dopamine Receptor D2 in Bridging the Intention-Behavior Gap in Sport Participation

**DOI:** 10.3390/ijerph18052379

**Published:** 2021-03-01

**Authors:** Seiyeong Park, Junhye Kwon, Chiyoung Ahn, Hae-Sung Cho, Hyo Youl Moon, Chung Gun Lee

**Affiliations:** Department of Physical Education, College of Education, Seoul National University, Seoul 08826, Korea; pseiy09@snu.ac.kr (S.P.); 2017_25059@snu.ac.kr (J.K.); chiyoung@snu.ac.kr (C.A.); 201826888@snu.ac.kr (H.-S.C.); skyman19@snu.ac.kr (H.Y.M.)

**Keywords:** A1 allele, DRD2, integrated behavioral model, sport participation, university students

## Abstract

Previous studies have identified that a behavior can occur through the strongest predictor intention, but there is a gap between intention and behavior. Dopamine receptor D2 (DRD2) is known to account for a variance in sporting behaviors in human and animal subjects. However, the relationship between DRD2 and sport participation has been poorly studied, and the limited available reports are inconsistent. The present study was performed to examine the impact of DRD2 on sport participation among Korean university students based on the integrated behavioral model (IBM). Data were collected from enrolled university students in Seoul (*N* = 45). Participants answered survey questions first, and then they gave investigators their hair to provide DNA information (i.e., the A1 allele of DRD2). DRD2 had a significant effect on sport participation, but only in male students. Male students who carried the A1 allele of DRD2 significantly participated in 105.10 min more sporting activities than male students who did not. Moreover, the effect of intention on sport participation was significantly decreased when considering DRD2. Despite the small sample size, the results of this study could be a preliminary case for a larger study and indicate the direction of future research. Our results suggest that DRD2 may have played an important role as the “actual skill” shown in the IBM.

## 1. Introduction

Physical inactivity is a global leading risk factor for non-communicable diseases and has a negative effect on quality of life and mental health [1,2]. Even though regular physical activity can help prevent health threats among various age groups, physical inactivity among college students in different countries was high (41.4%), and epidemiological evidence has shown that the level of physical activity steeply declines during college years [3,4]. College students decide their behaviors themselves, and they own responsibility for those behaviors [5]. It is important for college students to participate in regular physical activity because the behaviors that start in collegiate periods are often kept for a long time as habits [6,7].

Physical activity can be driven by participating in sporting activities. Participants can interact, compete, or achieve goals during sporting activities, and they can obtain the same benefits as physical activity, including increased muscle mass, improved self-esteem and body image, and reduced risk of obesity through sport participation [8,9]. Fortunately, regular sport participation among Korean university students has been increasing in recent years; however, it still looks unstable [10]. For these reasons, it is necessary to identify factors influencing decisions and choices concerning sport participation among college students.

Studies of participation in sporting activities have increasingly identified innate biological mechanisms as influencing factors along with psychosocial and environmental factors [11,12,13]. Regarding sport participation, both twin studies and family-resemblance models have indicated that a tendency to behave is genetically transmitted [14,15,16]. Most studies of family resemblance have shown a moderate correlation with heritability of sport participation (approximately 0.25), and genetics and environmental factors both appear to contribute significantly to participation in sport among twins [17]. The differential heritability of sport participation between genders remains ambiguous, although there is some evidence explaining the difference [15,16,17]. For example, genetic factors explained more than 80% of sport participation in male students, whereas environmental influences accounted for more variance in sport participation among female students than genetic factors [12].

Previous studies have found that dopamine receptor D2 (DRD2) accounts for variance in sporting behaviors in human and animal subjects [18,19,20]. DRD2 regulates the activities of humans by controlling the release of dopamine, which is a hormone influencing motivation and rewarding behaviors. Affected people seek out stimuli to reward themselves against reduced dopamine activities because when the A1 allele of DRD2 is increased, the level of DRD2 is decreased, resulting in a reward deficiency [21,22,23]. However, the relationship between genetic variance and sport participation has been poorly studied and cross-sectional study design was frequently used for the verification. Moreover, the limited available reports are inconsistent [14,24,25]. Among cross-sectional studies, Jozkow et al. (2013) found no relationship between sport participation and dopamine receptors D2 and D4 in Polish men [24]. Simonen et al. (2003) found that DRD2 had a significant association with sport participation; however, the results were applied only among women [20]. Unlike them, Lee et al. (2020) mitigated the uncertain causal problems previous studies had by investigating the effect of dopamine receptor genes on sport participation using a longitudinal approach [26]. Their findings have been confirmed only for male students, but it contributed to find the long-term effect of DRD2 on sport participation.

**Figure 1:**
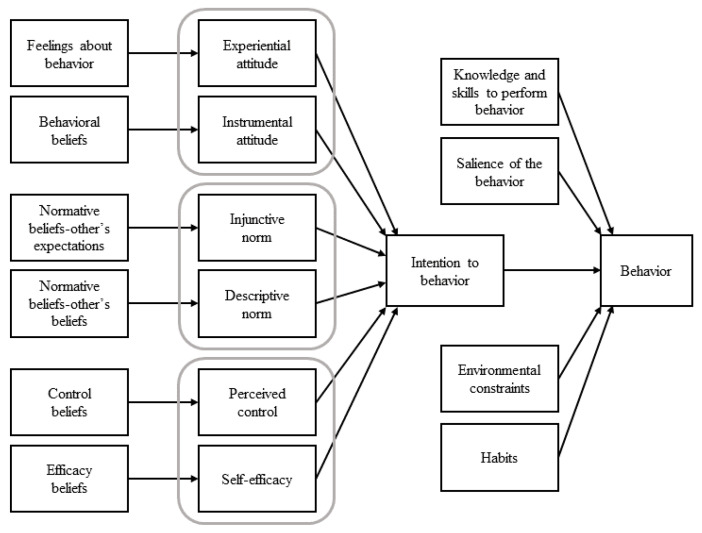
The integrated behavioral model [29].

The integrated behavioral model (IBM), developed from the theory of planned behavior, shows that behaviors can occur through the strongest predictor intention, but there are four other variables that explain behaviors directly (shown in Figure 1). These variables are measured objectively, unlike the subjectively measured variables predicting intention in the theory of planned behavior (i.e., attitude, subjective norm, and perceived behavioral control). Intention and behavior have low correlation and poor predictability in the theory of planned behavior, and the gap between intention and behavior has been a difficult problem to solve [27,28].

However, four components have been identified to explain the gap between intention and behavior in the IBM: knowledge and skills (i.e., actual skills), the salience of the behavior, environmental constraints, and habit [30,31]. They argued that a behavior can be accomplished if a person has the abilities required to perform it, the behavior is salient, environmental constraints to the behavior can be overcome, or the behavior is performed habitually. Thus, participation in sports can be maintained by setting goals to achieve in the sport or planning specific activities related to it using knowledge and skills [32,33,34]. It may be possible to fill the knowledge gap between intention and behavior by elucidating the influence of genetic factors on participation in sports. People having the A1 allele of DRD2 participate in sporting activities regularly through a mechanism of dopaminergic systems, which control motor movement in the brain. The dopaminergic system is related to rewarding and motivational behaviors, and can influence people to have concrete plans to participate in sports on their own through addiction to sports [35]. Lee et al. (2020) reported that adolescents with the A1 allele of DRD2 participate in sports more frequently, and that this habit is sustained until adulthood [26]. This suggests that A1 allele of DRD2 influences the performance of certain actions as an “actual skill” in the IBM.

Based on the literature, we focused on the genetic effect on sport participation as a potential influencing factor in the gap between intention and behavior, as suggested by the IBM. Furthermore, we analyzed data by gender separately based on previous studies that identified different effects of dopamine receptor genes on sport participation.

## 2. Materials and Methods

### 2.1. Participants and Design

The present study was designed to examine the relationship between dopamine receptor genes and sport participation based on the IBM. Participants were randomly selected from undergraduate departments in a university located in Seoul, South Korea. The total number of participants was 55, and both men and women participants were evenly distributed by grade levels. For the final analysis, only 45 participants (26 men and 19 women) were included because 7 subjects with missing values in the dependent variable were kept out, and 3 subjects were excluded based on regression diagnostics. Data were collected in October 2018 to avoid extremely cold or hot weather in South Korea and to avoid mid-term and final examinations in the university because students can be limited in their participation in sports. All protocols of this study were approved by the Seoul National University Institutional Review Board (IRB No.1810/003-016).

### 2.2. Measures

We used the questionnaire to identify the characteristics of students, their intentions to participate in sport, and their actual participation in sport. Students were asked about their intention to participate in sport using Ajzen’s three questions, as follows [36]: “Do you intend to participate in sport within a month?”, “Are you going to make efforts to participate in sport within a month?”, and “Do you have a plan to participate in sport within a month?” Each item was scored on a 7-point scale (−3 = strongly disagree to 3 = strongly agree). These three questions were averaged to calculate participants’ intention to participate in sport. The reliability of intention was calculated using Cronbach’s alpha coefficient (α = 0.93). To assess sport participation, students were asked two questions: “how many times have you participated in sport per week?” (e.g., three times a week) and “how much time have you participated in sport for each participation?” (e.g., 120 min at once). Then we multiplied the two values to calculate the weekly time spent participating in sporting activities.

### 2.3. Procedures

The authors trained investigators to conduct surveys and to collect genetic data. The educated investigators explained the purposes of our study, the procedures for collecting data, and the use of the results to the participants. Participants were given a packet that included consent forms that asked for agreement to respond to survey questions and provide genetic information, a survey questionnaire, and an incentive worth $10. Participating students answered survey questions first and then were asked to provide us with three or four strands of their hair—they gave investigators their hair for identification of the A1 allele of Taq1A polymorphism (rs1800497) of DRD2. The A1 allele of Taq1A polymorphism (rs1800497) is known to have an association with a reduction in novelty-seeking behavior, decreased reward sensitivity, and reduced DRD2 density in the striatum [26]. Hairs provided by participants were sealed in antiseptic containers with consequent numbers and moved to the laboratory. Investigators put their hairs in a 1.5 mL centrifuge tube. Samples were treated with 200 µL of E-prep reagent (Viagen Biotech, Inc., Los Angeles, CA, USA) and 0.5 µL proteinase K (Sigma-Aldrich Corporation, St. Louis, MO, USA). Those treated samples were incubated in a heating block at 56 °C for an hour and then incubated in a water bath at 85 °C for 45 min. They were centrifuged at 12,000 rpm for 3 min, and around 50 µL of supernatant were moved to another 1.5 mL tube. The estimated concentration of gDNA in each sample was quantified by NanoDrop (Thermo Fisher Scientific, Inc., Waltham, MA, USA) before performing polymerase chain reaction (RT-PCR). For DRD2 rs1800497 genotyping, the TaqMan Allelic Discrimination assay system (Thermo Fisher Scientific, Inc.) was used following the instructions given by Viagen Biotech, Inc. Twenty milliliters of total reaction volume, containing 90 ng of gDNA with nuclease-free water, 0.25 µL of primer-TaqMan Probe mixture, and 10 µL of TaqMan Universal PCR Master Mix 2X (Thermo Fisher Scientific, Inc) was used for genotyping. The PCR amplification conditions were as follows: 50 °C for 2 min, 95 °C for 20 s, followed by 111 cycles at 95 °C for 3 s, and 60 °C for 30 s using a CFX96 Real-Time PCR Detection System (Bio-Rad, Hercules, CA). Genotypes were determined using the relative fluorescence units (RFU) value under the single threshold algorithm in Maestro software (Bio-Rad).

### 2.4. Statistical Analysis

We combined the genetic data with the survey data to investigate the effect of DRD2 on the probability of sport participation of the participants. A t-test was conducted to compare the differences between the participants’ grades in school, and a chi-square test was used to compare determine the differences between groups of genes. Regression analysis was performed to examine the effects of the intention to participate in sport and the A1 allele of DRD2 on sport participation. All assumptions of multiple regression were tested. Since the homoscedasticity assumption was violated in male students, three outliers in the residual plot were deleted from the data. All statistical analyses in the present study were conducted using SAS version 9.4 (SAS Institute Inc., Cary, NC, USA).

## 3. Results

### 3.1. Descriptive Statistics

Table 1 shows the descriptive statistics of all participant data by gender. The number of male students was 34, and their grades in school were distributed evenly. According to our data, about one-third of male participants had at least one A1 allele of DRD2. The mean value of intention to participate in sporting activities was 1.71 (*SD* = 1.34) in male students, and their average sport participation time was 311.38 (*SD* = 281.08) minutes per week. Twenty-one female students participated in our study, and seniors accounted for the largest percentage among them. Similar to the male students, about 30 percent of the female students had one or more A1 allele of DRD2. In both genders there were no significant differences in grades or possession of the A1 allele. Female students’ intention to participate in sporting activities was 1.62 (*SD* = 1.02) on average, and their average sport participation time was 175.0 (*SD* = 169.45) minutes per week.

### 3.2. The Effect of A1 Allele of DRD2 on Sport Participation

The effects of DRD2 on sport participation among students are shown in Table 2. According to our results, intention had significant effects on sport participation in both male (model 1: coef = 69.51, *p* = 0.003; model 2: coef = 67.03, *p* = 0.002) and female students (model 1: coef = 165.50, *p* = 0.002; model 2: coef = 179.24, *p* = 0.010). The influence of DRD2 was found to significantly affect sport participation only among male students (coef = 105.10, *p* = 0.029). Male students who carry the A1 allele of DRD2 significantly participated in sporting activities 105.10 min more than male students who do not carry the A1 allele, and the effect of intention to participate in sport was significantly decreased when considering DRD2 in model 2. This means the A1 allele of DRD2 contributes to some of the effect of the intention of sport participation among male students. Unlike male students, a significant relationship was not shown between DRD2 and sport participation in female students in this result, and a significant interaction effect between intention and DRD2 was not found in either gender (not presented in the table).

## 4. Discussion

The present study found out that the intention to participate in sport had a significant influence on sport participation in both genders, and DRD2 had a significant effect on sport participation in male students only. The results that intention had a significant influence on sport participation and the effect of intention on sport participation was significant even after controlling for DRD2 indicate that intention is still important for predicting sport participation in Korean college students, even though previous reports have pointed to the inconsistency between intention and behavior [30,37].

More surprisingly, DRD2 had a significant effect on sport participation only in male students, according to our data. Male students with the A1 allele of DRD2 participated in sporting activities significantly more (about 105.10 min per week) than students without the A1 allele. Although the relationship was not significant in female students, the genetic effects on male students indicate that dopaminergic determinants should be considered when designing sport programs in the future. Our results are consistent with those of Lee et al. (2020), where the effects of dopamine receptor genes (DRD2, DRD4, and DRD5) on sport participation trajectories were revealed using longitudinal analysis [26]. They found that male students with the A1 allele of DRD2 participated significantly more in sporting activities and maintained these activities over time. However, more research will be required to examine gender differences in effects of DRD2 on sport participation according to gender, because there have been contradictory results in previous studies between genders [20,24].

The present study showed that the effect of intention on sport participation was decreased when controlling for DRD2 in male participants, even though the effects of intention were significant regardless of the possession of the A1 allele of DRD2 in both genders. This result indicates that DRD2 has the potential ability to narrow the gap between intention and sport participation among Korean university students. Although our results were different between male and female students, DRD2 may have played an important role as the “actual skill” shown in the IBM. Since increased extracellular dopamine concentration is known to motivate people to engage in certain behaviors, male students with the A1 allele of DRD2 may participate more in sport by setting specific goals and plans to participate in sporting activities than those who do not have the A1 allele [38,39].

However, DRD2 did not affect sport participation among female students. Some societies create a situation in which women must choose between participating in sports and maintaining a feminine image. This kind of conflict can destabilize the feminine identity of women, and therefore negate the life-long benefits of sport participation [40]. Since the traditional patriarchal system yet remains in South Korea, such ideological constraints can limit women’s opportunities for sport participation [41]. In our study, the effect of DRD2 on sport participation among female students may be weakened by ideological constraints (i.e., forcing women to choose sexually desirable images). This result suggests that it is important to consider environmental factors when investigating the effects of genes on behaviors.

Based on the findings of our study and the literature, we can say DRD2 is able to close existing loopholes between intention and behavior. The fact that students who have the A1 allele of DRD2 participated more in sport than others shows that DRD2 effectively elucidated sport participation of Korean college students. However, the result that the interaction effect between intention and DRD2 was not significant requires future studies to find the significant relationship between them. Moreover, our data showed there was a large difference in the sport participation time of students with the A1 allele of DRD2. Among those carrying the A1 allele of DRD2 in their brain, 51.52% of participants were placed between students who did not exercise at all and those who did more than 300 min a week. They are anticipated to get more involved in sport because their behavior is related to dopamine functioning. The A1 allele is known to be associated with DRD2 inhibitory function, regulating dopamine synthesis and release. Decreased DRD2 could lead to enhanced dopamine production and increases of extracellular dopamine concentrations. Increased amounts of dopamine in the brain bring people possessing A1 allele to greater risks of addictive behaviors, and people can feel a sense of reward and reinforcement through the behaviors [23,35]. If people experience it while playing sports, they become obsessed with higher addiction, and therefore, they will yearn for sport repeatedly.

Behavioral addictions can be caused by rational beliefs through cognitive reconstruction to impede upon social obligation [42]. College students are generally exposed to stressful situations from social relationships with other people, conflicts with professors or community members, or the establishment of newly discovered self-identity and self-esteem [43]. Especially, Korean university students endure excessive competition since they are traditionally anticipated to have outstanding academic achievement in order to attain a higher position in a given society [44]. This stressful situation can make them burned out and addicted to negative behaviors to avoid the social pressure. Sport has an attractive property that brings people an escape from serious issues in their normal life [45]. Even though sport can also cause negative outcomes like other addictions, it can prevent students from being exposed to severe dangers created by more extremely negative behaviors [26].

The present study has several limitations. First of all, the sample size of our data was small because many students refused to provide their genetic information for this study. Despite its small sample size, it has originality in that it is the first study that examined a genetic role in the sport participation of Korean college students. Secondly, we used a cross-sectional design to analyze data. The majority of studies related to genetic effects on sporting behaviors use a cross-sectional design, but it would be more effective to make people sustain sport participation if it were possible to conduct a longitudinal study with genetic data. Thirdly, sport participation was measured by self-reported questionnaires. This may have caused response bias or recall bias. Moreover, participants were not asked about the types of sport they participate in most. Future studies should obtain more specific information regarding the types of sport in order to better understand the influence of dopamine receptor genes on sport participation.

Notwithstanding the above-mentioned limitations, our findings provide critical evidence that dopamine receptor genes are important features for sport participation as the “actual skill” among Korean university students. According to the integrated behavioral model and our results, genetic factors might function as predictors of sport participation. Especially, dopamine receptor genes seem to be an important actual skill among Korean university students. Despite the lack of proven results, we propose that future researchers consider genetic factors in order to improve the predictive power of theories and narrow the intention–behavior gap. The accumulation of these studies will be able to provide crucial information when developing sport behavior promotion programs or policies.

## 5. Conclusions

To our knowledge, this is the first study attempting to narrow the gap between intention and sport participation using genetic information. The present research may contribute to the literature by giving important information, suggesting that DRD2 may play an important role as the “actual skill” of sport participation among Korean university students. Despite limitations, the results of this study would be a preliminary case by providing an opportunity for future researchers to consider genetic information as a potential predictor of sporting behavior based on behavioral theories and indicate the direction for the development of effective programs for promoting sport participation.

## Figures and Tables

**Table 1:** Descriptive statistics.

Characteristics	Males (*N* = 34)	Females (*N* = 21)	*p*-Value
Grade (%)			0.877
Freshman	7 (20.59)	5 (23.81)	
Sophomore	9 (26.47)	4 (19.05)	
Junior	8 (23.53)	4 (19.05)	
Senior	10 (29.41)	8 (38.80)	
DRD2 (%)			0.464
A1(−) group	21 (61.76)	15 (71.43)	
A1(+/++) group	13 (38.24)	6 (28.57)	
Intention			
Mean (*SD*)	1.71 (1.34)	1.62 (1.02)	<0.001
Sport participation			
Mean (*SD*)	311.38 (281.08)	175.0 (169.45)	<0.001

Note: DRD2, dopamine receptor D2; *SD*, standard deviation. A1(−) group includes participants who do not possess A1 allele of DRD2; A1(+/++) group includes participants who possess one or two A1 allele of DRD2.

**Table 2:** The effects of intention and the A1 allele of DRD2 on sport participation, and the interactions between variables.

	Model 1		Model 2	
	Estimate (*SE*)	*p*-Value	Estimate (*SE*)	*p*-Value
Males (*N* = 26)				
Intercept	104.84 (67.58)	0.135	73.32 (63.34)	0.260
Grade	6.96 (18.19)	0.706	−3.22 (17.22)	0.853
Intention	69.51 (20.62)	0.003	67.03 (18.91)	0.002
DRD2			105.10 (45.07)	0.029
Females (*N* = 19)				
Intercept	−17.96 (88.23)	0.841	12.18 (126.34)	0.925
Grade	−40.12 (27.23)	0.160	−50.28 (40.77)	0.237
Intention	165.50 (44.39)	0.002	179.24 (60.74)	0.010
DRD2			−36.78 (107.25)	0.736

Note: *SE*, standard error; DRD2, dopamine receptor D2. Model 1 is the grade-, and intention-controlled model; Model 2 is the model controlled for grade, intention, and DRD2.
